# Impact Of Discrimination In Healthcare On Patterns Of Doctor Visits Over Time

**DOI:** 10.1093/geroni/igaf122.1656

**Published:** 2025-12-31

**Authors:** Michael Green, Qing Yang, Hanzhang Xu, Radha Dhingra, Heather Farmer, Roland Thorpe, Matthew Dupre

**Affiliations:** Duke University School of Medicine, Durham, North Carolina, United States; Duke University, Durham, North Carolina, United States; Duke University, Durham, North Carolina, United States; Duke University School of Medicine, Durham, North Carolina, United States; University of Delaware, Newark, Delaware, United States; Johns Hopkins Bloomberg School of Public Health, Baltimore, Maryland, United States; Duke University School of Medicine, Durham, North Carolina, United States

## Abstract

Discrimination in healthcare negatively affects patient care. We examined how perceived discrimination in healthcare settings influenced long-term patterns of doctor visit among 17,642 Black and White middle-aged and older adults (ages 50-80 at baseline), using data from the 2008-2020 Health and Retirement Study. Discrimination in healthcare was assessed by asking participants how often they experienced poorer treatment from doctors or hospitals. Group-based trajectory models identified longitudinal patterns of doctor visits over 14 years of follow-up. Multinomial logistic regression models assessed whether perceived discrimination at baseline was associated with different trajectories of subsequent doctor visits, and whether sociodemographic factors, health behaviors, and health status attenuated the associations. We identified 5 trajectories of doctor visits: Low (∼2 visits/year; 33.5%), Frequent (∼4 visits/year; 33.1%), Frequent-to-High (∼4.5 visits/year increasing to ∼9; 13.7%), High-to-Frequent (∼13.5 visits/year decreasing to ∼6; 10.3%), and High (∼14 visits/year decreasing to ∼11; 6.3%). Groups with more doctor visits had poorer health, greater social vulnerability, and higher reported discrimination. Discrimination was associated with significantly greater risks of exhibiting trajectories with High (relative risk ratio [RRR]=1.62; P<.0001) and High-to-Frequent (RRR=1.38; P<.001) utilization relative to Low utilization after accounting for participants’ background factors. Overall, we found discrimination in healthcare was associated with trajectory groups exhibiting high doctor visits at baseline that subsequently declined. This suggests that among middle-aged and older adults with intensive healthcare needs, patient disengagement is more likely after experiencing discrimination. Health systems should consider the quality of care for patients with greater healthcare needs.

